# Associations of Systematic Inflammatory Markers with Diet Quality, Blood Pressure, and Obesity in the AIRWAVE Health Monitoring Study

**DOI:** 10.2147/JIR.S459238

**Published:** 2024-05-18

**Authors:** Ghadeer S Aljuraiban, Rachel Gibson, Linda M Oude Griep

**Affiliations:** 1 Department of Community Health Sciences, College of Applied Medical Sciences, King Saud University, Riyadh, 11451, Saudi Arabia; 2 Department of Nutritional Sciences, King’s College London, London, SE1 9NH, UK; 3 MRC Epidemiology Unit, University of Cambridge, Cambridge, CB2 0QQ, UK

**Keywords:** inflammatory markers, diet quality, blood pressure, obesity

## Abstract

**Introduction:**

Chronic low-grade inflammation is a characteristic feature of obesity, and elevated levels of inflammation are associated with pathophysiologic consequences and a constellation of metabolic disturbances, such as hypertension. The relationships of inflammation with diet, obesity, and hypertension are complex, hence, this study aimed to assess cross-sectional relationships between inflammatory scores, diet quality, obesity, high blood pressure (BP), and hypertension in the Airwave Health Monitoring Study cohort, a large cohort of police officers and police staff in the United Kingdom.

**Methods:**

Data from 5198 men and 3347 women who completed health screening measurements and dietary assessment between 2007 and 2012 were included (n=8545 adults). Platelet-to-lymphocyte ratio (PLR), neutrophil-to-lymphocyte ratio (NLR), lymphocyte-to-monocyte ratio (LMR), and the systemic immune-inflammation index (SII) were calculated. Diet quality was evaluated using the Nutrient-Rich Food 9.3 (NRF9.3) index score.

**Results:**

Results show that a 1SD higher diet quality score, waist circumference, and systolic/diastolic BP were significantly associated with SII differences of −33.3 (95% confidence interval (CI): −49.0, −17.6), 8.2 (95% CI: 0.2, 16.6), 17.9 (95% CI: 10.1, 25.8), and 18.3 (95% CI: 10.8, 25.7) (Model 2; P<0.0001), respectively. A 1SD higher diet quality score, waist circumference, and BMI were also significantly associated with PLR (P<0.0001). The odds of elevated PLR were higher in those with higher systolic and diastolic BP (P<0.0001, P=0.0006, respectively).

**Conclusion:**

In conclusion, the findings of this analysis add to the existing knowledge indicating a link between inflammation and conditions such as obesity, hypertension, and behavioral factors including diet quality. Of the various inflammatory scores evaluated, SII and PLR were consistently significantly associated with diet quality and these conditions.

## Introduction

The impact of diet on obesity and hypertension has been well described,^1–3^ and the adverse impact of obesity and hypertension is a global public health priority.^4^,^5^ The relationships between obesity, hypertension, inflammation, and diet are complex. Obesity promotes inflammation, and inflammation perpetuates the metabolic consequences of obesity.^6^ Chronic low-grade inflammation is a characteristic feature of obesity, and elevated levels of inflammation are associated with pathophysiologic consequences and a constellation of metabolic disturbances, such as hypertension.^6^

Recent studies showed that independent inflammation markers were associated with health outcomes and their risk factors, including cardiovascular disease^7^ and hypertension.^8^ Systemic inflammation can be evaluated from various biochemical markers widely available from routine blood tests, which provides a low-cost alternative to other measures of inflammation (eg, interleukin-6, TNF-α).^9^ Since these markers are influenced by various factors including hydration level and exercise,^10^ ratios derived from these markers were created to measure inflammation and immune status with higher stability and predictability including platelet-to-lymphocyte ratio (PLR),^10^ neutrophil-to-lymphocyte ratio (NLR),^7^ lymphocyte-to-monocyte ratio (LMR),^9^ and the systemic immune-inflammation index (SII).^11^ Although reference levels of these prognostic inflammation markers in healthy populations are not well established yet, population-specific reference intervals were developed to enhance the prognostic capabilities of these markers^12–17^ that can serve as relative indicators of inflammation within a population with higher PLR, NLR, and SII scores and lower LMR scores usually indicative of poorer prognoses of diseases.^7^,^10^,^11^,^18^

Although inflammation ratios were found associated with specific health conditions in patient populations,^19–23^ there is so far limited research on these ratios in generally healthy populations. Moreover, research has indicated that high intakes of saturated and trans-fatty acids, high glycemic foods, extra virgin olive oil, and low intakes of vitamin D and a low magnesium status may influence inflammation markers,^22–30^ but research evaluating overall diet quality and its influence on these inflammation ratios is an emerging area of research. For instance, a recent cross-sectional study of middle-aged to older adults in Ireland found higher diet quality or less inflammatory diets were associated with lower levels of NLR and other inflammatory biomarkers.^31^ This same cohort was also used to demonstrate that the healthfulness of a plant-based diet and diet quality determined using the Nutri-Score rating was associated with NLR and other inflammatory biomarkers.^32^,^33^ Additionally, diet quality relates to inflammation via weight gain and obesity,^34^ this underpins the importance of taking into account the influence of obesity on these associations.

Therefore, the current study assesses cross-sectional relationships between inflammatory scores (ie, SII, PLR, LMR, NLR) and diet quality, obesity, blood pressure, and hypertension in the Airwave Health Monitoring Study, a large-scale epidemiological cohort of police officers and police staff in the United Kingdom (UK)^35^ with detailed food records and anthropometric and biochemical measurements required to investigate these relationships.^35^

## Materials and Methods

### Study Design and Procedures

The Airwave study details were reported in detail.^35^ Briefly, in 2004, members of the British police force were enrolled in the study reaching (n= 53,114) participants by 2015. For the current cross-sectional analysis, participants who completed health screening measurements and dietary assessment between 2007 and 2012 were included (n= 9018). We excluded participants diagnosed with diabetes or CVD, and those with extremely low or high energy intake (<500 kcal/d or >5000 kcal/d for women or 8000 kcal/d for men^36^ (n=473). The final sample included (n=8545) adults (5198 men and 3347 women). Airwave participants were informed about the purpose of the study and written consent to participate was provided by all participants. The study protocol was approved by the National Health Service Multi-Site Research Ethics Committee (MREC/13/NW/0588).

### Socio-Demographic Characteristics

During the health screening visits, trained personnel conducted clinical examinations for each participant following a standardized protocol. Socio-demographic and lifestyle data (eg, age, education level, and smoking) were collected using a self-administrated online questionnaire. The short version of the International Physical Activity Questionnaire was used to assess physical activity. Participants were asked to report the frequency and duration of specific activities in Metabolic Equivalent of Task (MET)-minutes/week, and based on this data, levels of physical activity (high, moderate, or low) were assigned.^37^

### Biochemical Data

Blood samples were collected from each participant and transported to the laboratory. Quality assurance and control of all laboratory equipment were frequently carried out. Hematological parameters including platelet count and differential white cell count (neutrophils, lymphocytes, monocytes) were obtained using a hematology analyzer and related reagents (Siemens Advia 2120), in accordance with the instructions. The SII, PLR, NLR, and LMR were calculated as follows:

$${\mathit{SII = }}{{{{\rm Platelet\ count }}\left({{{\rm 1}}{{{\rm 0}}^{{\rm 9}}}{{\rm /L}}} \right){{\rm \times\ Neutrophil\ count }}\left({{{\rm 1}}{{{\rm 0}}^{{\rm 9}}}{{\rm /L}}} \right)} \over {{{\rm Lymphocyte\ count }}\left({{{\rm 1}}{{{\rm 0}}^{{\rm 9}}}{{\rm /L}}} \right)}}$$

$${\mathit{PLR = }}{{{{\rm Platelet\ count }}\left({{{\rm 1}}{{{\rm 0}}^{{\rm 9}}}{{\rm /L}}} \right)} \over {{{\rm Lymphocyte\ count }}\left({{{\rm 1}}{{{\rm 0}}^{{\rm 9}}}{{\rm /L}}} \right)}}$$

$${\mathit{NLR = }}{{{{\rm Neutrophil\ count }}\left({{{\rm 1}}{{{\rm 0}}^{{\rm 9}}}{{\rm /L}}} \right)} \over {{{\rm Lymphocyte\ count }}\left({{{\rm 1}}{{{\rm 0}}^{{\rm 9}}}{{\rm /L}}} \right)}}$$

$${\mathit{LMR = }}{{{{\rm Lymphocyte\ count }}\left({{{\rm 1}}{{{\rm 0}}^{{\rm 9}}}{{\rm /L}}} \right)} \over {{{\rm Monocyte\ count }}\left({{{\rm 1}}{{{\rm 0}}^{{\rm 9}}}{{\rm /L}}} \right)}}$$

### Dietary Data

Participants reported their dietary intake using a 7-day food diary. Photographs and common household measures developed by Nelson et al were used to estimate portion sizes more accurately.^38^ Participants were given instructions to provide details on cooking methods, brand names, and portion sizes. Trained dietitians followed the study operational manual for coding the diaries and matching recorded food/beverage items to a UK Nutritional database code and a portion size.^39^ Nutrient analysis was done using Dietplan software (version 6.7; Forestfield Software Ltd., Horsham, UK) using the UK food composition database of McCance and Widdowson.^40^ Quality control measures were taken to ensure accuracy and consistency.

### Nutrient-Rich Food 9.3 Index-Score

To evaluate the quality of the diet, the Nutrient-Rich Food 9.3 (NRF9.3) index score was used.^41^ This score is highly correlated with the Healthy Eating Index 2005, which is a measure of the quality of diet recommended by the US Dietary Guidelines.^42^ The NRF9.3 index score is calculated by summing percentages of daily nutrient intakes of nine nutrients that are beneficial for health (protein, dietary fiber, vitamins A, C, E, calcium, iron, potassium, and magnesium) and then subtracting the sum percentages of maximum recommended values of three nutrients that should be limited (saturated fat, added sugar, and sodium) per 100 kcal. A higher NRF9.3 index score indicates a higher nutrient quality per 100 kcal.

### Anthropometric Measurements

Weight and height were measured twice during the health screening visit while participants wore light clothes and without shoes with a weighing scale and a portable stadiometer (Marsden H226). The body mass index (BMI) was calculated by dividing the weight (kg) by the square of the body height (m). Waist-circumference was also measured twice at the mid-axillary line using a measuring tape.

### Blood Pressure Measurement

Blood pressure (PB) was measured three times, 30 seconds apart, with participants seated and relaxed using (Omron HEM 705-CP, OMRON Corp., Kyoto, Japan). Having a systolic BP (SBP)) ≥140 mmHg or diastolic BP (DBP) ≥90 mmHg^43^ or self-reporting a diagnosis of hypertension or taking antihypertensive medication was defined as having hypertension.

### Statistical Analysis

SAS version 9.3 by SAS Institute in Cary, NC, USA, was utilized for the statistical analysis. Any result with a p-value <0.05 was deemed statistically significant. Baseline characteristics of participants were presented as mean (SD) or %, stratified by gender.

To ensure our analysis was reliable, we first checked for normality of the data by examining the skewness of residuals. We also evaluated homoscedasticity by reviewing plots of residuals against predicted values.^44^ After assumptions were satisfied, we checked for multicollinearity using type 2 tolerance where a value less than 0.10 indicated collinearity.^45^ Afterward, we applied multivariate linear regression models adjusted for potential confounders to identify associations with SII, PLR, NLR, and LMR for each 1 SD higher NRF9.3 index score (8.9), BMI (4.1 kg/m^2^), waist circumference (11.9 cm), SBP (15.3 mmHg), and DBP (10 mmHg). Model 1 was adjusted for age, sex, and employment country, while Model 2 was additionally adjusted for marital status, education, ethnicity, annual household income, smoking, alcohol intake, and medication use.

Logistic regression analysis was used to estimate the odds of elevated SII, PLR, NLR, and LMR by quartiles and per 1 unit increase of NRF, BMI, and BP. The 80^th^ percentile of each marker served as cutoff values for detecting an elevated inflammatory marker.^46^

Potential effect modification by age, sex, and BMI was checked using interaction terms and stratified analysis by sex and BMI. The odds of having elevated PLR, NLR, and LMR per 1 unit increase in NRF, BMI, waist circumference, and BP were also investigated in stratified analysis by age groups.

## Results

### Baseline Characteristics

The sample included 8545 participants (5198 men and 3347 women), who were mostly of white ethnicity (98%) and had an average age (±SD) of 41.0 (±9.2) years (Table 1). The average BMI was 27.0 (±4.1) kg/m^2^, which was higher in men than women [27.8 (±3.6) vs 25.8 (±4.6) kg/m^2^ respectively] and the mean waist circumference was 89.2 (±11.9) cm. Average SBP was 130.9 (±15.3) mmHg, with higher readings in men than women [135.8 (±13.7) vs 123.2 (±14.3) mmHg respectively], while average DBP was 79.6 (±10.0) mmHg. As for diet quality, the mean NRF9.3 index score was 19.5 (±8.9), which was higher in women than in men (21.2 (±9.7) vs 18.4 (±8.1)), respectively.

**Table 1:** Characteristics Stratified by Gender, n=8545

	Men		Women		Total
*n*	5198		3347		8545
Age (y)	42.1	(8.8)	39.4	(9.5)	41.0 (9.2)
White ethnicity (%)	97.2		98.0		97.5
Marital status (%)					
Cohabiting	13.5		19.9		16.0
Divorced/separated	6.5		17.8		10.9
Married	6.5		9.8		7.8
Single	72.1		48.8		63.0
Missing	1.4		3.7		2.3
Education (%)					
Left school before taking GCSE	4.3		3.2		3.9
GCSE or equivalent	31.1		28.0		29.9
Vocational qualifications	7.1		7.1		7.1
A levels / higher or equivalent	32.0		32.0		32.0
Bachelor’s degree or equivalent	19.9		22.6		21.0
Postgraduate qualifications	5.6		7.0		6.2
Annual household income (%)					
Less than £26,000	3.8		15.3		8.3
£26,000–£37,999	16.2		21.8		18.4
£38,000–£57,999	44.7		32.7		40
£58,000–£77,999	25.3		20.1		23.3
More than £ 78,000	10.0		10.0		10.0
Employment (force) country (%)					
England	70.8		74.0		72.1
Scotland	18.3		13.5		16.4
Wales	9.4		10.4		9.8
Missing	1.5		2.1		1.7
Smoking status (%)					
Current	6.7		9.8		7.9
Former (<12 months)	24.0		23.4		23.7
Never or quit (≥12 months)	69.3		66.9		68.4
Alcohol intake (%)					
Current	93.7		89.9		92.2
Former (<12 months)	4.4		6.7		5.3
Never or quit (≥12 months)	1.9		3.4		2.5
Systolic BP (mmHg)	135.8	(13.7)	123.2	(14.3)	130.9 (15.3)
Diastolic BP (mmHg)	81.6	(9.7)	76.5	(9.6)	79.6 (10.0)
Hypertension (%)	39.3		17.0		30.0
BMI (kg/m^2^)	27.8	(3.6)	25.8	(4.6)	27.0 (4.1)
Waist-circumference (cm)	93.9	(9.6)	81.7	(11.3)	89.2 (11.9)
Overweight (%)	78.4		49.4		66.9
Obesity (%)	22.9		15.2		19.8
Total cholesterol (mg/dL)	207.3	(39.6)	197.2	(38.1)	203.4 (39.3)
C reactive protein (mg/dL)	1.7	(2.6)	2.2	(3.3)	1.9 (2.9)
Platelets	248.9	(55.6)	274.9	(61.7)	259.1 (59.4)
Lymphocytes	1.7	(0.5)	1.8	(0.5)	1.8 (0.5)
Neutrophils	3.7	(1.3)	4.1	(1.4)	3.9 (1.4)
Monocytes	0.4	(0.2)	0.4	(0.2)	0.4 (0.2)
White blood cells	6.3	(1.5)	6.7	(1.8)	6.4 (1.7)
SII	576.0	(325.4)	660.6	(329.7)	609.1 (329.7)
PLR	152.8	(54.2)	160.6	(53.5)	155.8 (54.1)
LMR	4.5	(1.6)	5.4	(1.9)	4.9 (1.8)
NLR	2.3	(1.1)	2.4	(1.0)	2.3 (1.0)
Nutrients					
Total energy (kcal)	2048	(474.9)	1652	(380)	1893 (481)
Carbohydrates (% of total energy)	46.9	(7.0)	48.4	(7.0)	46.5 (7.0)
Protein (% of total energy)	18.2	(3.2)	17.7	(3.2)	17.2 (3.2)
Fat (% of total energy)	34.9	(5.4)	33.9	(5.6)	33.9 (5.5)
NRF9.3 index score	18.4	(8.1)	21.2	(9.7)	19.5 (8.9)
NRF9.3 index score individual components (per 1000 kcal)					
Protein (g)	43	(8)	43	(8)	43 (8)
Fiber (g)	9	(3)	10	(3)	9 (3)
Vitamin A (IU)	1465	(1191)	1733	(1397)	1570 (1280)
Vitamin E (mg)	4	(1)	4	(1)	4 (1)
Vitamin C (mg)	44	(27)	56	(32)	49 (30)
Calcium (mg)	451	(108)	470	(115)	458 (110)
Magnesium (mg)	152	(30)	155	(33)	153 (31)
Iron (mg)	6	(2)	6	(2)	6 (2)
Potassium (mg)	1621	(320)	1720	(372)	1660 (345)
Saturated fatty acid (g)	14	(3)	14	(3)	14 (3)
Total sugar (g)	47	(14)	51	(15)	48 (15)
Total sodium (mg)	1447	(312)	1447	(339)	1447 (322)

**Note**: Data are presented as mean (SD) or %.

**Abbreviations**: BMI, Body mass index; BP, Blood pressure; LMR, lymphocytes-monocyte ratio; NLR, neutrophil-lymphocytes ratio; NRF, nutrient rich food index; PLR, platelet-lymphocyte ratio; SII, systemic immune-inflammation index.

The average SII was 609.1 (329.7), higher in women (660.6 (329.7)) than in men (576.0 (325.4)). Mean PLR, LMR, and NLR were as follows; 155.8 (54.1), 4.9 (1.8), and 2.3 (1.0), respectively, all higher in women compared to men.

### Relation Between Inflammatory Markers and Diet Quality, Obesity Indices, and BP

**Figure 1:**
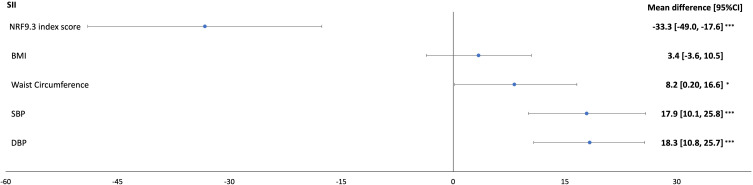
Estimated mean difference in SII associated with a 1-SD higher NRF9.3 index score, BMI, waist circumference, SBP, DBP in a sample of the Airwave Health Monitoring Study (n=8545). Model 1 is adjusted for age, sex, and employment country. Model 2 is model 1 adjusted for marital status, education, ethnicity, annual household income, and alcohol intake.1 SD in NRF9.3 index score (8.9), BMI (4.1), waist circumference (11.9), SBP (15.3), DBP (10). *p <0.05; ***p < 0.0001.

**Abbreviations**: BMI, body mass index; DBP, diastolic blood pressure; NRF, nutrient rich food index; SII, systemic immune-inflammation index; SBP, systolic blood pressure.

A 1SD higher NRF9.3 index score was significantly associated with an SII difference of −33.3 (95% CI: (−49.0, −17.6)) and a PLR difference of −4.4 (95% CI: −7.0, −1.8) (Model 2; Table 2 and Figure 1). There were no significant associations found between the NRF9.3 index score and NLR or LMR.

**Table 2:** Estimated Mean Difference in SII, PLR, NLR, LMR Associated with a 1-SD Higher NRF9.3 Index Score, BMI, Waist Circumference, SBP, DBP in a Sample of the Airwave Health Monitoring Study (n=8545)^a^

	SII	PLR	NLR	LMR
	Mean Difference (95% CI)	Mean Difference (95% CI)	Mean Difference (95% CI)	Mean Difference (95% CI)
**NRF9.3 index score**				
Model 1	−36.9 (−52.5, −21.3)	−3.6 (−6.2, −1.1)	−0.1 (−0.1, 0.1)	0.07 (−0.01, 0.10)
Model 2	−33.3 (−49.0, −17.6)	−4.4 (−7.0, −1.8)	−0.1 (−0.1, 0.1)	0.05 (−0.01, 0.10)
**BMI**				
Model 1	4.6 (−2.5, 11.6)	−6.1 (−7.2, −4.9)	0.1 (0.1, 0.1)	0.12 (−0.02, 0.26)
Model 2	3.4 (−3.6, 10.5)	−6.0 (−7.1, −4.8)	0.1 (−0.1, 0.1)	0.12 (−0.02, 0.26)
**Waist Circumference**				
Model 1	9.9 (1.6, 18.3)	−6.2 (−7.6, −4.9)	0.1 (0.1, 0.1)	0.12 (−0.02, 0.26)
Model 2	8.2 (0.2, 16.6)	−6.0 (−7.4, −4.6)	0.1 (−0.1, 0.1)	0.12 (−0.02, 0.26)
**SBP**				
Model 1	18.5 (10.6, 26.4)	−1.0 (−2.3, 0.3)	0.1 (0.1, 0.1)	0.03 (−0.05, 0.02)
Model 2	17.9 (10.1, 25.8)	−1.0 (−2.3, 0.3)	0.1 (0.1, 0.1)	0.03 (−0.05, 0.02)
**DBP**				
Model 1	18.9 (11.5, 26.3)	−0.5 (−1.7, 0.7)	0.1 (0.1, 0.1)	0.10 (−0.04, 0.04)
Model 2	18.3 (10.8, 25.7)	−0.5 (−1.7, 0.8)	0.1 (0.1, 0.1)	0.10 (−0.04, 0.04)

**Notes**: ^a^Model 1 is adjusted for age, sex, and employment country. Model 2 is model 1 adjusted for marital status, education, ethnicity, annual household income, and alcohol intake 1 SD in NRF9.3 index score (8.9), BMI (4.1), waist circumference (11.9), SBP (15.3), DBP (10).

**Abbreviations**: BMI, body mass index; DBP, diastolic blood pressure; LMR, lymphocytes-monocyte ratio; NLR, neutrophil-lymphocytes ratio; NRF, nutrient rich food index; PLR, platelet-lymphocyte ratio; SII, systemic immune-inflammation index; SBP, systolic blood pressure.

**Figure 2:**
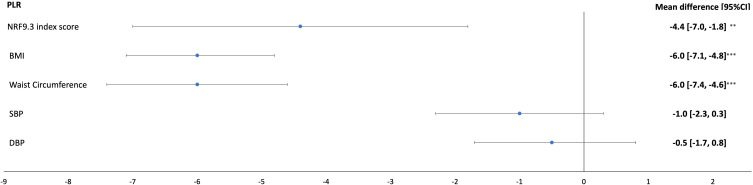
Estimated mean difference in PLR associated with a 1-SD higher NRF9.3 index score, BMI, waist circumference, SBP, DBP in a sample of the Airwave Health Monitoring Study (n=8545). Model 1 is adjusted for age, sex, and employment country. Model 2 is model 1 adjusted for marital status, education, ethnicity, annual household income, and alcohol intake.1 SD in NRF9.3 index score (8.9), BMI (4.1), waist circumference (11.9), SBP (15.3), DBP (10). **p < 0.001; ***p < 0.0001.

**Abbreviations**: BMI, body mass index; DBP, diastolic blood pressure; NRF, nutrient rich food index; PLR, platelet-lymphocyte ratio; SBP, systolic blood pressure.

BMI was inversely associated with PLR only (−6.0, 95% CI: −7.1, −4.8). Waist circumference however was directly associated with SII (8.2, 95% CI: 0.2, 16.6), but inversely related to PLR (−6.0, 95% CI: −7.4, −4.6) (Figure 2).

For BP, there were direct associations observed between SBP and DBP with SII; 17.9 (95% CI: 10.1, 25.8), and 18.3 (95% CI: 10.8, 25.7), respectively.

There was no significant association between diet quality and the odds of elevated inflammatory markers. Higher odds of elevated PLR, however, were observed among the higher quartiles of BMI (p for trend<0.0001) (Model 2, Table 3). There was also a significant trend between higher waist circumference and the odds of elevated SII (p=0.01), and PLR (p<0.0001). Similarly, across quartiles of SBP and DBP, the odds of elevated SII increased with higher SBP and DBP (p<0.0001, p=0.0006, respectively) (Model 2, Table 3).

**Table 3:** Odds Ratio of Elevated SII, PLR, NLR, and LMR by Quartiles and per 1 Unit Increase of NRF, BMI, SBP, DBP in a Sample of the Airwave Health Monitoring Study (n=8545)^a,b^

	Q1	Q2	Q3	Q4	P for Trend	NRF by 1SD
**NRF, median score**	>24.2	18.4–24.2	13.5–18.4	<13.5		8.9
Cases	1601/2140	1632/2142	534/2141	554/2139		2137/6408
Elevated SII model 1	1.00	0.99 (0.87–1.15)	1.08 (0.94–1.24)	1.17 (1.01–1.34)	0.09	0.99 (0.99, 1.00)
Model 2	1.00	0.99 (0.87–1.15)	1.08 (0.94–1.24)	1.14 (0.99–1.31)	0.21	0.92 (0.98, 0.99)
Elevated PLR model 1	1.00	1.08 (0.94–1.25)	0.94 (0.82–1.09)	1.02 (0.89–1.18)	0.32	0.99 (0.99, 1.00)
Model 2	1.00	1.06 (0.92–1.22)	0.93 (0.81–1.07)	1.01 (0.88–1.16)	0.33	0.99 (0.99, 1.00)
Elevated NLR model 1	1.00	0.98 (0.86–1.14)	1.05 (0.91–1.20)	1.07 (0.94–1.23)	0.63	0.99 (0.99, 1.00)
Model 2	1.00	0.98 (0.86–1.14)	1.04 (0.91–1.19)	1.06 (0.92–1.21)	0.75	0.99 (0.99, 1.00)
Elevated LMR model 1	1.00	0.97 (0.84–1.12)	1.05 (0.89–1.21)	1.01 (0.89–1.17)	0.78	1.00 (0.99, 1.01)
Model 2	1.00	0.97 (0.84–1.12)	1.04 (0.90–1.21)	1.00 (0.87–1.16)	0.83	1.00 (0.99, 1.01)
	**Q1**	**Q2**	**Q3**	**Q4**	**P for trend**	**BMI by 1SD**
**BMI, median score**	<24.2	26.6–24.2	29.3–26.6	>29.3		4.1
Cases	579/2153	524/2136	500/2137	534/2136		2136/6409
Elevated SII model 1	1.00	1.06 (0.92–1.22)	1.07 (0.93–1.24)	1.13 (0.98–1.10)	0.40	1.00 (0.99, 1.00)
Model 2	1.00	1.05 (0.91–1.21)	1.05 (0.90–1.21)	1.10 (0.95–1.27)	0.64	1.00 (0.99, 1.02)
Elevated PLR model 1	1.00	0.68 (0.59–0.78)	0.72 (0.62–0.83)	0.91 (0.80–1.05)	<0.0001	0.95 (0.94, 0.96)
Model 2	1.00	0.67 (0.58–0.78)	0.71 (0.62–0.83)	0.91 (0.80–1.05)	<0.0001	0.95 (0.94, 0.97)
Elevated NLR model 1	1.00	0.91 (0.79–1.05)	0.88 (0.76–1.01)	0.88 (0.77–1.02)	0.24	0.99 (0.98, 1.00)
Model 2	1.00	0.91 (0.79–1.04)	0.85 (0.74–0.99)	0.91 (0.79–1.04)	0.11	0.99 (0.98, 1.00)
Elevated LMR model 1	1.00	1.09 (0.94–1.26)	1.09 (0.93–1.26)	0.99 (0.86–1.15)	0.47	1.01 (0.99, 1.02)
Model 2	1.00	1.09 (0.94–1.26)	1.09 (0.94–1.26)	1.00 (0.87–1.16)	0.45	1.01 (0.99, 1.02)
	**Q1**	**Q2**	**Q3**	**Q4**	**P for trend**	**Waist circumference by 1SD**
**Waist circumference, median score**	<80.95	80.95–89.00	89.00–96.95	>96.95		11.9
Cases	520/1680	487/1589	552/1620	578/1536		
Elevated SII model 1	1.00	1.22 (1.06–1.42)	1.28 (1.09–1.51)	1.33 (1.13–1.56)	0.003	1.00 (0.99, 1.01)
Model 2	1.00	1.21 (1.04–1.40)	1.25 (1.07–1.47)	1.29 (1.09–1.51)	0.01	1.00 (0.99, 1.02)
Elevated PLR model 1	1.00	1.03 (0.89–1.19)	0.90 (0.77–1.05)	1.03 (0.89–1.19)	<0.0001	0.98 (0.98, 0.99)
Model 2	1.00	1.03 (0.89–1.19)	0.91 (0.77–1.06)	1.03 (0.89–1.19)	<0.0001	0.98 (0.98, 0.99)
Elevated NLR model 1	1.00	1.04 (0.90–1.20)	0.96 (0.82–1.13)	0.91 (0.77–1.06)	0.27	0.99 (0.98, 1.00)
Model 2	1.00	1.02 (0.89–1.18)	0.94 (0.80–1.09)	0.87 (0.74–1.02)	0.15	0.99 (0.98, 1.00)
Elevated LMR model 1	1.00	0.94 (0.82–1.08)	1.03 (0.88–1.21)	0.92 (0.78–1.08)	0.39	1.01 (0.99, 1.02)
Model 2	1.00	0.93 (0.79–1.10)	1.04 (0.88–1.22)	0.94 (0.82–1.09)	0.45	1.01 (0.99, 1.02)
	**Q1**	**Q2**	**Q3**	**Q4**	**P for trend**	**SBP by 1SD**
**SBP, median score**	<120	120–129.7	129.7–140	>140		15.3
Cases	533/1591	565/1577	514/1644	525/1613		
Elevated SII model 1	1.00	0.74 (0.64–0.86)	0.75 (0.65–0.87)	0.69 (0.59–0.80)	<0.0001	1.01 (1.00, 1.02)
Model 2	1.00	0.74 (0.64–0.86)	0.76 (0.65–0.88)	0.69 (0.59–0.80)	<0.0001	1.01 (0.99, 1.01)
Elevated PLR model 1	1.00	0.90 (0.78–1.04)	0.89 (0.77–1.04)	0.87 (0.74–1.01)	0.28	1.00 (0.97, 1.03)
Model 2	1.00	0.91 (0.78–1.04)	0.90 (0.78–1.05)	0.88 (0.75–1.02)	0.36	1.00 (0.97, 1.00)
Elevated NLR model 1	1.00	0.87 (0.76–1.01)	0.89 (0.76–1.03)	0.87 (0.75–1.01)	0.19	1.00 (0.99, 1.01)
Model 2	1.00	0.88 (0.76–1.01)	0.90 (0.78–1.04)	0.89 (0.76–1.03)	0.28	1.00 (0.99, 1.01)
Elevated LMR model 1	1.00	1.04 (0.90–1.19)	1.02 (0.88–1.18)	1.08 (0.92–1.26)	0.79	1.00 (0.99, 1.01)
Model 2	1.00	1.03 (0.89–1.19)	1.00 (0.86–1.17)	1.06 (0.91–1.24)	0.87	1.00 (0.99, 1.01)
	**Q1**	**Q2**	**Q3**	**Q4**	**P for trend**	**DBP by 1SD**
**DBP, median score**	<72.3	72.3–79	79–85.7	>85.7		10
Cases	532/1606	503/1624	533/1588	569/1607		
Elevated SII model 1	1.00	0.99 (0.86–1.14)	0.85 (0.73–0.99)	0.76 (0.66–0.88)	0.0003	1.01 (0.99, 1.01)
Model 2	1.00	0.99 (0.86–1.15)	0.86 (0.74–0.98)	0.76 (0.66–0.88)	0.0006	1.01 (1.00, 1.02)
Elevated PLR model 1	1.00	0.94 (0.82–1.08)	0.95 (0.82–1.09)	0.91 (0.79–1.05)	0.62	1.00 (0.99, 1.00)
Model 2	1.00	0.95 (0.82–1.09)	0.96 (0.83–1.11)	0.92 (0.79–1.05)	0.68	1.00 (0.99, 1.00)
Elevated NLR model 1	1.00	0.99 (0.84–1.11)	0.86 (0.75–0.99)	0.95 (0.82–1.09)	0.15	1.00 (0.99, 1.01)
Model 2	1.00	0.98 (0.85–1.12)	0.87 (0.76–1.00)	0.99 (0.84–1.12)	0.22	1.00 (0.99, 1.01)
Elevated LMR model 1	1.00	1.05 (0.91–1.20)	1.00 (0.87–1.15)	1.12 (0.97–1.29)	0.40	1.00 (0.99, 1.01)
Model 2	1.00	1.04 (0.91–1.20)	0.98 (0.87–1.15)	1.10 (0.97–1.29)	0.44	1.00 (0.99, 1.01)

**Notes**: ^a^Data are presented as OR (95% CI). ^b^Model 1 is adjusted for age, sex, and employment country. Model 2 is model 1 adjusted for marital status, education, ethnicity, annual household income, and alcohol intake.

**Abbreviations**: BMI, Body mass index; BP, Blood pressure; LMR, lymphocytes-monocyte ratio; NLR, neutrophil-lymphocytes ratio; NRF, nutrient rich food index; PLR, platelet-lymphocyte ratio; SII, systemic immune-inflammation index.

### Relation Between Inflammatory Markers and Diet Quality, Obesity Indices, and BP in Sub-Groups

There was no indication of potential effect modification by age, sex, BMI, BP, or diet quality, and despite that, we conducted stratified analysis using Model 2 (Table S1). Results remained statistically significant between NRF9.3 index score and SII for participants with overweight, normal-weight, healthy SBP/DBP, elevated HTN, those 30 to ≤40 years of age, and those 40 to ≤50 years of age. Results however attenuated in participants with poor/neutral/healthy diet quality, obese participants, those with elevated SBP, ≤30 years of age, and >50 years of age. We found similar findings for PLR. There were no significant results between the NRF9.3 index score and NLR and LMR.

For BMI, results were significant for SII in normal-weight participants only. For PLR, significance was not changed for all subgroups. For waist circumference, the association with SII prevailed for obese participants, those younger than 30 years, and those between 30–40 years of age. Similar findings were observed for SBP where the significance of results for SII remained unchanged for participants with neutral diet quality, obese participants, overweight participants, normal weight participants, those with elevated SBP/DBP, ≤30 years of age, 30 to ≤40 years of age, and 40 to ≤50 years of age (Table S1). There was no significant association between diet quality, obesity indices, and BP with the odds of elevated inflammatory markers subgroups (Table S2).

## Discussion

### Main Findings

The present study evaluated the cross-sectional relationships between inflammatory scores (ie, SII, PLR, LMR, NLR), diet quality, obesity, hypertension, and high blood pressure in the Airwave Health Monitoring Study cohort. The results demonstrated significant inverse associations of diet quality with SII and PLR and of BMI/waist circumference with PLR. Significant positive associations were observed for waist circumference and BP with SII. No associations were found for any of the lifestyle factors with either LMR or NLR. While the presented results correspond with previous research that demonstrated comparable associations between inflammation and obesity, hypertension, high blood pressure, and diet, differential relationships between specific inflammatory scores and these factors were found, as discussed below.

### Inflammatory Scores and Diet Quality

The inverse relationship between inflammatory scores and diet quality concurs with previously reported findings. For example, the anti-inflammatory potential of the Mediterranean diet has been reported in a systematic review of cross-sectional studies where participants with a higher Mediterranean diet score had lower levels of c-reactive protein, TNF-α, fibrinogen, and interleukin-6.^26^ Further, a meta-analysis of dietary intervention trials found that greater adherence to the Mediterranean diet was inversely associated with c-reactive protein, interleukin-6, and intracellular adhesion molecule-1.^47^ In addition, a large cross-sectional cohort of over 14,000 people found that platelet and white blood cell counts were inversely related to adherence to the Mediterranean diet.^48^ The NRF 9.3 index, used to measure diet quality in the current study, highly correlates with the US Healthy Eating Index (HEI).^42^ In a cross-sectional study, a higher HEI-2015 score was associated with lower levels of inflammatory biomarkers (ie, c-reactive protein, interleukin 6) and white blood cell counts among 46–73 year-olds.^49^ Specific to the markers examined in the present work, poor diet quality and unhealthy plant-based diets were positively associated with NLR^32^,^33^, and a less pro-inflammatory diet was associated with lower NLR^31^ in recent cross-sectional analyses of Irish middle-to-older age adults. This differs from the findings of our work which found no associations with NLR.

### Inflammatory Scores and Obesity

We found that only PLR was significantly associated with BMI. This is consistent with other research that found non-significant associations between some inflammatory scores and obesity, though the specific score differed by study. For instance, from cross-sectional studies, Ryder et al found no relationship between NLR and BMI,^50^ Lin et al found no relationship between BMI and NLR and PLR among men using data from a longitudinal study of twins,^51^ and Furuncuoǧlu et al found a relationship between SII with BMI but not with PLR, NLR, and other biomarkers not investigated in the present work.^52^

Though we found no indication of potential effect modification by sex, we observed that SII was higher in women than in men [660.6 (329.7) vs 576.0 (325.4)]. Several factors could potentially contribute to the observed sex-specific differences in SII. Hormonal differences, specifically estrogen and progesterone, play a significant role in regulating immune responses and inflammation.^53^ Variations in hormone levels during the menstrual cycle can influence inflammatory markers. For example, estrogen has anti-inflammatory effects, while progesterone can be both pro- and anti-inflammatory depending on the context.^53^,^54^ Another contributing factor is related to the distribution of adipose tissue between men and women, which can affect inflammation.^55^ Women tend to store more fat in the gluteal-femoral region, whereas men store more fat in the abdominal and visceral depot. In post-menopausal women, more fat is accumulated in the visceral depot. Fatty acid mobilization and oxidation can lead to different fat distribution between men and women.^54^ Furthermore, a few studies found that markers of inflammation, specifically C reactive protein, are strongly associated with measures of adiposity, and this correlation is generally stronger in women than in men.^55^,^56^ Our findings corroborate these results, as women had higher levels of C reactive protein than men [(2.2 (3.3) vs 1.7 (2.6) mg/dL].

### Inflammatory Scores and Blood Pressure

Another aspect of the present work that conflicts with our findings is the association between inflammatory scores (ie, SII and PLR) and hypertension. Inflammation can contribute to the development of hypertension through endothelial cell dysfunction, alterations in the gut microbiome, lipid oxidation, and more.^57^ The present findings coincide in general, but not completely with some previous research. A cross-sectional study of over 22,000 adults showed a positive association between SII and NLR and the prevalence of hypertension, but a negative relationship with PLR and LMR.^8^ In another study, NLR was associated with incident hypertension primarily among older adults and males, but not females, younger adults, and BMI-specific groups.^58^ Discrepancies between the findings of the present study and those previously conducted may be due to factors that may influence inflammatory biomarkers, such as specific health conditions in patient populations as well as heterogeneity between methodologies. Even with similar research questions, the variability in prognostic scores between populations limits the generalizability of findings to other groups.^12^

### Comparison Between Inflammatory Scores

With the exception of BMI, SII was associated with all the other factors examined in this study. Some studies suggest that SII has a stronger relationship with health outcomes when compared to the other inflammatory scores.^8^,^11^,^59^ For example, SII was found to have a stronger positive relationship with hypertension than NLR,^8^ SII prediction ability in terms of hepatocellular carcinoma was better than that of the NLR and PLR,^11^ and SII showed greater prognostic value than NLR, PLR, and MLR for patients with cervical cancer.^59^ This may be explained by the fact that SII incorporates three individual parameters, thus reflecting more aspects of the inflammatory response.

An important aspect to consider in interpreting our findings and comparing them to previous research or using them for prognostic purposes is the lack of established reference intervals for the scores. While some research has identified reference intervals, they are not necessarily generalizable to the current cohort. For example, the reference intervals established from a large Dutch population-based prospective cohort study of individuals ≥45 years found that 95% reference intervals for NLR, PLR, and SII were 0.83–3.92, 61–239, and 189–1168, respectively, which increased with age and differed by sex (eg, females had higher PLR and SII).^14^ In another study, Meng et al identified the reference intervals in Chinese adults (>18 years), which were different from those found in the Dutch cohort.^12^ In the same cohort, reference intervals of 161–701 for SII, 61–179, and 55–179 for PLR were identified for those ≤65 years and >65 years, 0.9–2.94 and 0.85–3.06 for NLR for males and females ≤65 years, 0.95–3.57 and 0.83–3.30 for NLR for males and females >65 years, 2.50–7.50 and 2.75–8.50 for LMR for males and females ≤65 years, 2.16–7.41 and 2.40–8.33 for LMR for males and females >65 years, 0.12–0.35 and 0.10–0.32 for MLR for males and females ≤65 years, and 0.12–0.41 and 0.11–0.33 for MLR for males and females >65 years.^12^ Since cutoffs for these inflammatory measures have not been established yet and considering the potential of a large variety in levels due to lifestyle factors and genetics,^13^ the numerical scores in the present analysis are currently only interpretable relative to other scores from this same cohort.

One finding of note is that the relationships between PLR and diet and PLR and waist circumference were found significant in many of the subgroups examined, except for people aged over 50 years. Meng et al found that PLR, NLR, LMR, and monocyte-lymphocyte ratio (MLR) were significantly different between age groups in China.^12^ Additionally, PLR and NLR increased with age in a study of Dutch twins, which was postulated to be due to the presence of underlying, but undiagnosed, diseases among older populations.^51^ Considering that many of these inflammatory scores appear to vary by age, this may explain our findings which need to be confirmed in future longitudinal research.

## Strengths and Limitations

There are several limitations of this work. The study was cross-sectional, and therefore, causal relationships could not be determined. Some variables used in this study, despite being accepted and commonly used methods of data collection, were self-reported and, thus, subject to recall or misreporting bias (eg, food diaries). The Airwave Health Monitoring Study was designed primarily to answer questions related to the use of Terrestrial Trunked Radio and health, not to investigate the relationship between inflammatory scores and diet quality, high blood pressure, and obesity.^35^ However, the data collected from all over the UK provides a valuable opportunity to examine health-related research questions.^35^,^60–65^ The results may be limited in terms of generalizability to the broader UK population or populations outside of the UK since only police officers and policing staff were included, primarily males with limited staff from ethnic minorities.^35^ As seen in other research, age and sex impact the reference intervals.^12^ The extent to which the results are applicable to the wider population is unknown, but biological pathologies may be similar to other groups. Future studies can assess the applicability of these findings to other populations, with the inclusion of unmeasured confounders, eg, pollution exposure^66^ and shift work.^67^,^68^ Additionally, it is worth noting that the Airwave Health Monitoring Study was designed as a long-term observational study with regards to TETRA exposure. However, this information was not available at the time of our analysis and TETRA and other related environmental variables will be investigated in a more comprehensive manner in a future study.

## Conclusions

A better understanding of the link between inflammatory scores, diet, hypertension, high blood pressure, and obesity, allows further understanding of the utility of these low-cost inflammation markers and their potential prognostic value for monitoring the anti-inflammatory potential of dietary patterns, risk stratification, and the population-specific relevance for assessing hypertension and obesity risk. Several inflammatory scores were examined in the current work with SII and PLR emerging as the two markers consistently associated with obesity, hypertension, high blood pressure, and dietary intake.
